# Switchable Negative Group Delay Based on Sandwich Topological Protection Structure in Terahertz Band

**DOI:** 10.3390/nano15040251

**Published:** 2025-02-07

**Authors:** Jiao Xu, Xianmin Pan, Jiao Tang, Xianghua Peng, Yuxiang Peng

**Affiliations:** 1 School of Information Science and Engineering, Hunan Women’s University, Changsha 410004, China; xujiao@hnwu.edu.cn (J.X.); panxianmin@hnwu.edu.cn (X.P.); 2 School of Physics and Electronics, Hunan Normal University, Changsha 410081, China; tangjiao0717@hunnu.edu.cn; 3 Hunan Province Key Laboratory of Materials Surface & Interface Science and Technology, Institute of Mathematics and Physics, Central South University of Forestry and Technology, Changsha 410004, China

**Keywords:** group delay, topological protection structure, terahertz band

## Abstract

A switchable enhancement group delay in the terahertz band based on a novel sandwich topology protection structure with graphene is proposed in this paper. The notable phase transition of the reflected beam comes from the topological edge-protected mode excited at the sandwich photonic crystal surface, and the non-trivial topology of the photonic crystal allows the structure to be immune against defects and imperfections, which lays the foundation for the enhancement of group delay in the terahertz band. And the introduction of graphene creates favorable conditions for the reversible switching of positive and negative reflection group delay. Moreover, the reflected group delay can also be flexibly and dynamically controlled by the incident angle. The positive and negative reversible switching reflected group delay proposed in the terahertz band greatly reduces the optical transmission loss and significantly increases the transmission efficiency compared with the traditional metal sandwich structure, which provides a feasible idea for the realization of multi-dimensional manipulation of the wavelength and phase of electromagnetic waves in the terahertz band. The novel scheme is expected to provide potential applications in fields such as optical buffers or ultrafast modulators.

## 1. Introduction

Terahertz waves are regarded as a potential development direction for sixth-generation (6G) communications and future optical communications because of their high frequency and large bandwidth [1,2]. This continued growth in demand for optical communications development continues to spur the development of subsystems that will be critical to future optical networks [3], such as optical buffers [4] and ultrafast modulators [5]. In recent years, group delay (GD) has been extensively investigated, such as infinite GD at a phase singularity [6], large GD based on Bragg grating [7,8], integrated GD for reconfigurable spectrum sensing of millimeter-wave signals [9], and large GD based on electromagnetically induced transparency metamaterial [10]. However, signal transmission in the terahertz band faces development bottlenecks such as serious signal attenuation and difficulty in long-distance transmission. Therefore, the design and exploration of GD devices with large numerical effects, low energy loss, and compact structural design in the terahertz band have become a focus of attention.

On the one hand, the local field enhancement effect can be stimulated by the design of specific structural modes and combined with novel materials with excellent optical properties to realize the enhancement of GD in the terahertz band. Conventional metal–insulator–metal (MIM) three-layer structures (often also referred to as sandwich structures) are applied regularly in the field of optical micro- and nanodevices research and preparation [11], but they are often accompanied by significant energy loss. With the continuous breakthroughs and innovations for the traditional structures, integrated silicon platforms with new phenomena and mechanisms have ushered in new development opportunities in the field of micro- and nanoscale GD device research. Photonic crystals (PhCs), which are periodic optical structures with the advantages of good light field confinement and multiple adjusting degrees of freedom, provide a powerful platform to control the flow of light. In addition, PhCs, which have the advantages of good light field confinement and multiple adjusting degrees of freedom, have been widely used to reveal different topological phases of light and demonstrate topological photonic functionalities [12]. Topological protection has an unprecedented and ideal transmission characteristic, which is independent of defects and backscattering, enabling lossless transmission of light [13,14], and thus has promising applications in topological photonics [15]. At the same time, the robust transport of photons promotes the development of photonic topological quantum information processing and quantum computation [16]. Non-trivial topology of the photonic crystal ensures backscattering-free light propagation around the path and allows the structure to be immune against defects and imperfections, which provides a feasible guarantee for the enhancement of GD [17]. Therefore, it is expected to combine topological photonics with conventional sandwich structures to propose a novel sandwich structure with topological protection modes, which is expected to realize terahertz GD enhancement with low loss and high transmission efficiency on conventional integrated silicon platforms.

On the other hand, the realization of effective manipulation of GD enhancement based on its realization in the terahertz band becomes an aspect that cannot be ignored. Two-dimensional materials show more flexible modulation and richer optical properties compared with traditional dielectric materials, thus creating favorable conditions for effective manipulation of GD. Among them, graphene, as a typical 2D material with excellent optoelectronic properties such as high bandwidth and low loss [18,19], has received favorable attention in GD. The graphene’s conductivity can be controllably flexible by applying an external voltage, and the manipulation of conductivity can affect the absorption of the composite system constructed by graphene and other materials, which will have a significant advantage in the control of GD. Therefore, controlling the transmission of optical pulse signals by controlling the conductivity of graphene [20,21] provides good conditions for realizing effective control of GD [22,23,24] and can be considered as a competitive solution for realizing tunable GD in the terahertz band.

In this paper, a novel sandwich topological protection structure with graphene is proposed for the realization of multidimensional manipulation of electromagnetic wave wavelength and phase within the terahertz band to stimulate the enhancement of tunable positive- and negative-switchable GD. The topological edge-protected states created by the sandwich structure consisting of two different types of photonic crystals allow the incident light to form a drastic phase change. Considering that almost all opto-mechanical systems are difficult to be tuned [25], flexible and richly diverse tuning of terahertz GD is realized by combining a novel sandwich topology protection structure with graphene, whose unique optical properties make the large positive GD excited by the novel sandwich topology protection structure to be reversed, forming a negative GD, and then continuously tuned with a controllable applied voltage. In addition, by adjusting parameters such as the number of graphene layers and the angle of incident light, the means of dynamically regulating terahertz GD can be further enriched. It is shown that the composite heterostructure composed of a novel sandwich topology protection structure and graphene can realize the dynamic modulation of positive and negative switching of the terahertz GD effect, which will provide a reliable solution for the research of optical buffers and ultrafast modulators, which are crucial for 6G communication and future optical communication.

## 2. Materials and Methods

### 2.1. Materials

**Figure 1:**
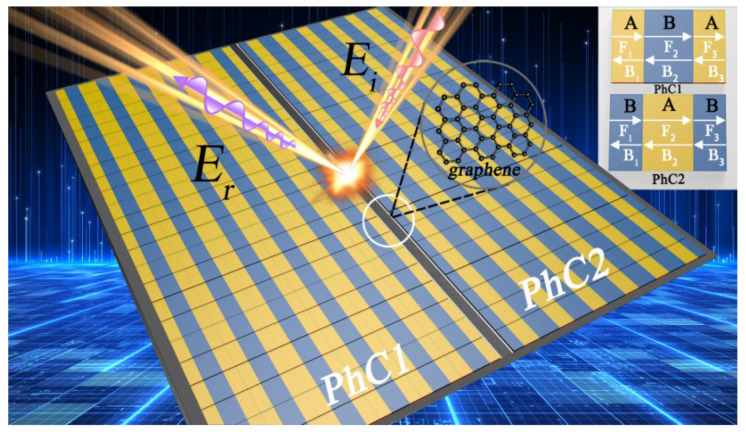
Schematic diagram of a topological protection structure at THz frequencies. The incidence angle is θ.

We consider a topological protection structure using two different PhCs. Specifically, we stack two PhCs belonging to different topological types together, which forms a novel variety of artificial collective modes, resulting in a one-dimensional superlattice band structure. Similar heterostructures have previously been presented in graphene nanoribbons and topological insulator superlattices [26]. As seen in Figure 1, the topological protection structure is composed of two kinds of PhCs (PhC-α and PhC-β) and defective cells D1 and D2. The refractive index is set to be *n* = 2 for medium A (HfO_2_) and *n* = 1.46 for medium B (SiO_2_). The thicknesses are $d_{A}=38.35\;\mu m$, $d_{B}=45\;\mu m$, and defective cells is d=5 μm. The unit cells of PhC-α and PhC-β are denoted as $PhC-\beta =A_{dA/2}B_{dB}A_{dA/2}$, $PhC-\beta =A_{dB/2}B_{dA}A_{dB/2}$. The defect unit D is denoted as $D_{1}=A_{d}$, $D_{2}=B_{d}$, The period of each PhC is here defined as T = 6, and thus the stacked structure consisting of repeating α(β)-type cells can be characterized as α6(β6). It is assumed that light is incident at angle θ and only TM polarization is considered in this paper.

### 2.2. Characterizations

Meanwhile, graphene is inserted into the surface between D1 and D2 to dynamically regulate GD. Considering that covalent bonds generally exist between the freestanding nanomembranes and host substrate, 2D graphene films can be layered and transferred into arbitrary rack optical structures without the need for expensive equipment and lengthy development processes required by epitaxial methods [27,28]. We use surface conductivity to characterize its dispersion relationship. The graphene’s isotropic surface conductivity $\sigma_{0}$ is the sum of the $\sigma_{inter}$ and the $\sigma_{intra}$, calculated as follows [29]:

(1) $$\sigma_{0}=\sigma_{inter}+\sigma_{intra},$$

(2) $$\sigma_{intra}=\frac{ie^{2}k_{B}T}{\pi h^{2}(\omega +\frac{i}{\tau })}(\frac{E_{F}}{k_{B}T}+2ln(e^{-\frac{E_{F}}{k_{B}T}}+1)),$$

(3) $$\sigma_{inter}=\frac{ie^{2}}{4\pi \hbar }ln\left|\frac{2E_{F}-(\omega +i\tau^{-1})\hbar }{2E_{F}+(\omega +i\tau^{-1})\hbar }\right|,$$

where e is electron charge, ħ is reduced Planck’s constant, τ is the relaxation time, $E_{F}=\hbar V_{F}\sqrt{\pi n_{2D}}$ is the Fermi energy, and $n_{2D}$ is the carrier density. Here, we set N = 1, T = 300 K, τ = 0.9 ps, and EF = 0.05 eV. It can be seen that the conductivity of graphene can be adjusted flexibly by controlling gate voltage, which can be used to dynamically regulate group delay. However, the inter-band conductivity tends to be ignorable for the THz frequencies range because of the photon energy $\hbar \omega \ll E_{F}$.

### 2.3. Calculations

The reflection coefficient and reflection phase can be obtained by the transfer matrix method, as can the transmission coefficient and phase. The propagation matrix can be expressed in terms of electromagnetic wave propagation in a medium, written as follows:

(4) $$P\left(d\right)=\left[\begin{array}{cc} e^{-ik_{z}d} & 0\\ 0 & e^{ik_{z}d} \end{array}\right],$$

The graphene’s conductivity can be reflected in the boundary conditions [30], so the transmission matrix can be expressed as for TM polarization, written as follows:

(5) $$D_{a\to a}=\frac{1}{2}\left[\begin{array}{cc} 1+\eta +\xi & 1-\eta -\xi \\ 1-\eta +\xi & 1+\eta -\xi \end{array}\right],$$

where $\eta =\varepsilon_{1}k_{2z}/\varepsilon_{2}k_{1z}$, $\xi =\sigma k_{2z}/\varepsilon_{0}\varepsilon_{2}\omega$, and k is the wave vector of light propagating through the medium. Apparently, ξ is strongly dependent on the graphene’s conductivity, which provides ideas for dynamically modulating the group delay.

Thus, GD can be expressed as follows, assuming the incident pulse is a Gaussian pulse [16]:

(6) $$\tau_{r}={\left[\partial \phi /\partial \omega \right]}_{\omega =\omega_{c}},$$

where $\phi_{r}$ is the phase and $\omega_{c}$ carrier frequency.

## 3. Results and Discussions

**Figure 2:**
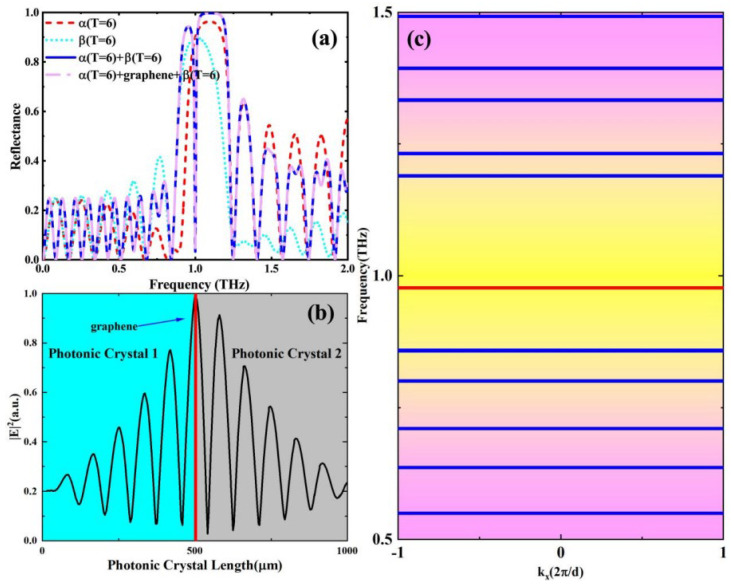
(**a**) The reflectance spectra of α-type (red, short-dashed line), β-type (cyan, short-dotted line), the “α+β” heterostructure (blue, solid line) and the “α+graphene+β” (pink, dash-dotted line); (**b**) Electric field distribution of topological sandwich structure; (**c**) The energy band of topological sandwich structure.

Figure 2 shows the reflectance, electic field distribution and band gap of the sandwich topological protection structure. The relationships of the incident frequency to the reflectance of α-type, β-type, “α+β”, and “α+graphene+β” are shown in Figure 2a. It can be observed that the optical topological states cannot be excited by individual α-type and β-type PhCs and that individual PhCs have a large photonic band gap around 1 THz, keeping the reflectivity at a high value. By constructing a sandwich topological protection structure, the drastic reflectance dip appears near 1 THz due to the fact that the emergence of the topological state is accompanied by an enhancement of the local field as shown in Figure 2b (which is obtained from COMSOL Multiphysics). The drastic reflectivity change makes it possible to obtain large GD near the resonance point as shown by Equation (6). By calculating the energy band of the topological sandwich structure, it can be proved that the TES mode is generated at the interface of α-type and β-type, and there exists a topological energy band with a frequency of 0.987 THz in the common energy gap 0.86 THz ~ 1.18 THz of the PhC1 or PhC2 structure, as shown in Figure 2c, which does not exist with single a PhC [31]. Concurrently, a single graphene sheet is interposed between photonic crystals in order to facilitate the dynamic modulation of GD. Here, we postulate a non-contact interface between the graphene and the dielectric surface; thereby, the graphene’s impact on GD predominantly manifests through the transmission matrix governing the interface of two PhCs. It can be deduced that minute alterations in the graphene’s conductivity suffice to effectuate modulation of GD. As can be seen from the purple dashed line in Figure 2a, the introduction of graphene can bring rich means for GD modulation while maintaining the reflective dip of this structure.

**Figure 3:**
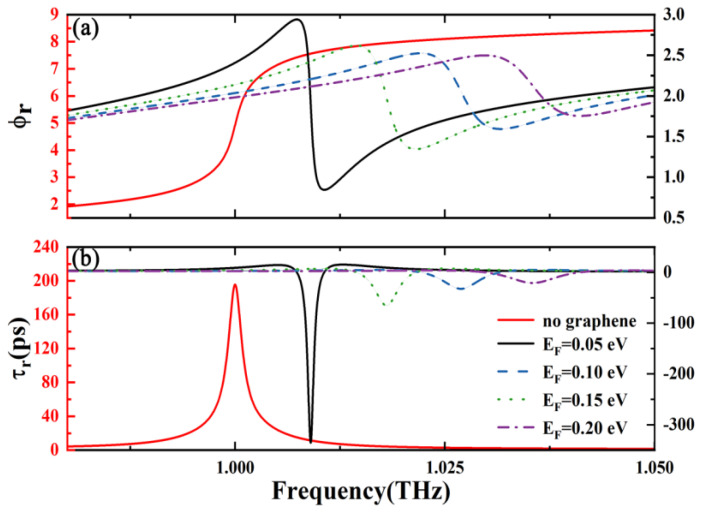
Dependence of the (**a**) reflected phase, $\phi_{r}$, and (**b**) reflected group delay, $\tau_{r}$, on frequency for different Fermi energies of graphene. For comparison, reflected phase and group delay without graphene is shown as well (red solid line).

Figure 3 shows the dependence of reflected phase and GD for different Fermi energies. It is assumed the electromagnetic wave incident is vertical. As depicted in Figure 3, the introduction of the topological edge state triggers a continuous phase change that has a very large slope within the PhC heterostructure. This leads to a significant reflected group delay (about 202 ps) in the absence of graphene. Furthermore, the conductivity of graphene is significantly influenced by the Fermi energy as indicated in Equations (1)–(3). Consequently, by manipulating the external voltage to alter the Fermi energy, one can dynamically adjust both the sign and the magnitude of GD. As shown in Figure 3, GD about −335 ps can be obtained when the Fermi energy is 0.05 eV, which is much larger than previous work [32]. And GD is still negative but with smaller values if the Fermi energy continues to increase. It can be observed that when the Fermi level is 0.10 eV, 0.15 eV, or 0.20 eV, the group delay is −66 ps, −32 ps, or −21 ps, respectively. In conclusion, it can be observed that GD before the introduction of graphene is positive, and in a certain range, it jumps to negative and even the absolute value is greater than before. Taking 0.05 eV as an example, it can be found that the absolute value increased by 41.8% compared to before the introduction of graphene. Additionally, these occurrences underscore the crucial role that the applied voltage assumes in dictating both the sign and magnitude of GD. By modulating the Fermi energy level through an external voltage, it becomes feasible to dynamically adjust the reflected GD within a stationary structure, offering a highly effective external control mechanism.

**Figure 4:**
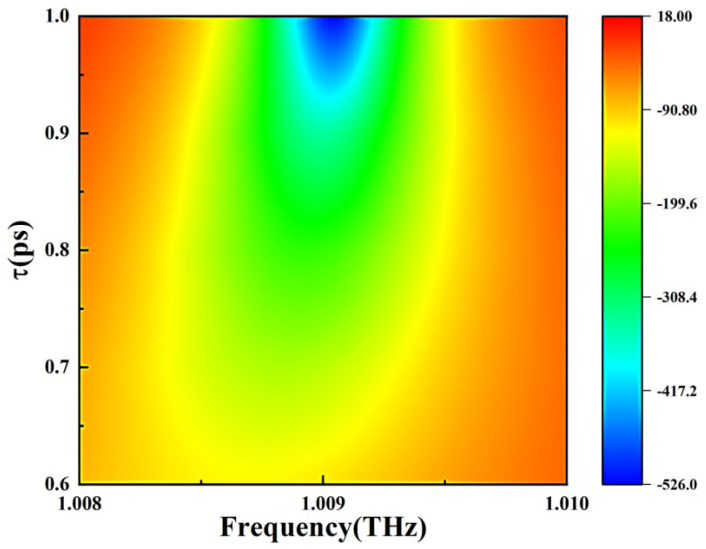
Dependence of the reflected group delay, $\tau_{r}$, on frequency for different relaxation times, τ, of graphene.

Figure 4 shows the dependence of reflected GD for different relaxation times. In particular, according to Equations (1)–(3), the conductivity is also affected by the relaxation times. Therefore, as shown in Figure 4, we calculate the effect of graphene relaxation time on the phase when the Fermi energy of graphene is a constant value of 0.05 eV, while keeping other parameters consistent with that of Figure 3. The change in graphene relaxation time increases the reflection phase slope and GD in the overall system but, interestingly, does not affect the central frequency of the group delay. The change of Fermi energy and relaxation time of graphene leads to different effects of GD, which provides a feasible scheme for the electrically tunable GD of the system.

**Figure 5:**
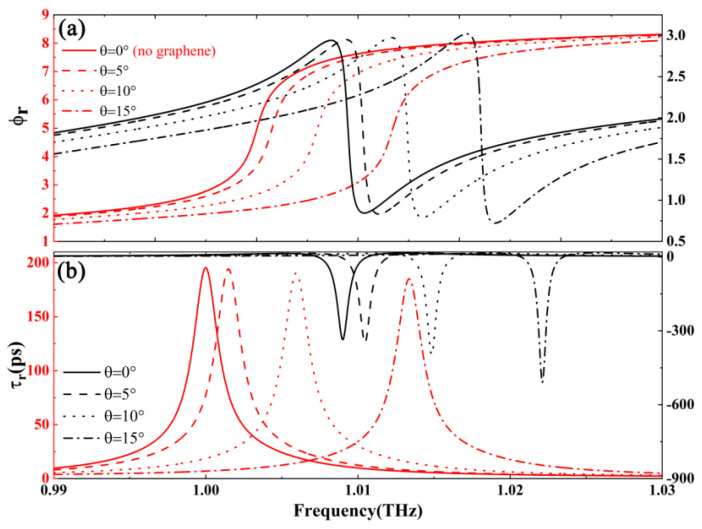
Dependence of the (**a**) reflected phase, $\phi_{r}$, and (**b**) reflected group delay, $\tau_{r}$, on frequency for different incident angles with graphene (black line), without graphene (red line).

Figure 5 shows the dependence of reflected GD for different incident angles. On the premise that the structural parameters and graphene parameters are consistent with the above figure, we consider whether the change of the incident angle will have a significant impact on the group delay of the system. As shown in Figure 5, the incident angle does not enable a flexible conversion between negative and positive group delays (also known as superluminal and subluminal) but has a significant effect on the value of the GD. For example, the reflected GD is about −335 ps when θ = $0^{{}^{\circ}}$. As the angle increases to θ = $15^{{}^{\circ}}$, the value of the group delay is about −508 (with graphene). And the change in the incident angle also affects the reflection group delay of the overall structure when graphene is not present. However, the effect on GD is significantly weaker than that in the presence of graphene in the composite structure. This is also related to the location of graphene in Figure 2, where the local electric field is enhanced, and the excellent optical properties of graphene have more obvious responses and changes to the regulation of the same degree of freedom.

**Figure 6:**
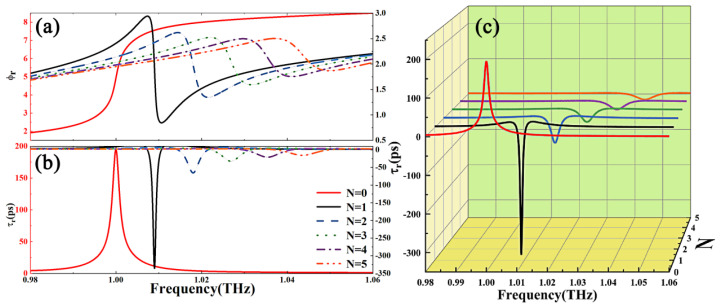
Dependence of the (**a**) reflected phase, $\phi_{r}$, and (**b**,**c**) reflected group delay, $\tau_{r}$, on frequency for different N layers of graphene.

Figure 6 shows the dependence of reflected GD for different numbers of layers of graphene. Based on this, the presence of graphene has an important impact on the regulation of GD in fixed structures. Considering that graphene is a single-layer, two-dimensional material, when the number of layers of graphene N≤5, its conductivity can be approximated to $\sigma \approx N\sigma_{0}$. We not only consider the phase transition changes affecting GD by changing the graphene parameters by applying voltage but also calculate the effect of changing the graphene structural parameters on the size of the reflection GD. As shown in Figure 6a, an increase in the number of graphene layers decreases the slope of the reflection phase and the value of GD. When N = 1, the reflection group delay is about −335 ps, which has the largest absolute value. When N = 5, the absolute value of reflection group delay decreases to about −16 ps. As shown in Figure 6b, the effect of a change in the number of graphene layers on reflection GD is significant.

## 4. Conclusions

In summary, we have investigated the enhancement of switchable GD in terahertz bands with a novel sandwich graphene topological protection structure. The topological edge-protected states consisting of two different types of photonic crystals in the novel sandwich topological protection structure allow the incident light to form a drastic phase change, which creates favorable conditions for the excitation of large positive GD. In addition, almost all opto-mechanical systems are difficult to tune, but the introduction of graphene, due to its excellent and unique optoelectronic properties, enables the reversal of the positive large group delay effect to form the negative group delay effect and provides a flexible and rich variety of schemes for the dynamic manipulation of the GD. Further, the positive-negative switchable GD of the novel sandwich topology-protected structure is also closely related to parameters such as the number of graphene layers and the angle of incident light. Our work gives sufficient theoretical analysis to verify the positive-negative switchable GD effect with the topological edge protection property. Compared with the traditional metal sandwich structure, the fabrication process requirements are relatively low, the transmission loss of terahertz waves is greatly reduced, and the transmission efficiency is significantly increased. These provide an excellent solution for the realization of multi-dimensional manipulation of wavelength and phase in the terahertz band and are expected to provide a potential application for optical buffers or ultrafast modulators in the subsystems that are crucial to the future optical networks and to improve the transmission efficiency of the whole optical network and communication system even further.

## Data Availability

The original contributions presented in this study are included in the article. Further inquiries can be directed at the corresponding author.
